# Dynamic changes of rhizosphere soil bacterial community and nutrients in cadmium polluted soils with soybean-corn intercropping

**DOI:** 10.1186/s12866-022-02468-3

**Published:** 2022-02-15

**Authors:** Han Li, Luyun Luo, Bin Tang, Huanle Guo, Zhongyang Cao, Qiang Zeng, Songlin Chen, Zhihui Chen

**Affiliations:** 1 Crop Research Institute, Hunan Academy of Agricultural Sciences, Changsha, China; 2 Yangtze Normal University, Chongqing, China

**Keywords:** Rhizosphere bacterial community, Monoculture, Intercropping, Soil available cadmium, Soil physicochemical properties, α diversity, Phylogenetic molecular ecological network, Illumina sequencing

## Abstract

**Background:**

Soybean-corn intercropping is widely practised by farmers in Southwest China. Although rhizosphere microorganisms are important in nutrient cycling processes, the differences in rhizosphere microbial communities between intercropped soybean and corn and their monoculture are poorly known. Additionally, the effects of cadmium (Cd) pollution on these differences have not been examined. Therefore, a field experiment was conducted in Cd-polluted soil to determine the effects of monocultures and soybean–corn intercropping systems on Cd concentrations in plants, on rhizosphere bacterial communities, soil nutrients and Cd availability. Plants and soils were examined five times in the growing season, and Illumina sequencing of 16S rRNA genes was used to analyze the rhizosphere bacterial communities.

**Results:**

Intercropping did not alter Cd concentrations in corn and soybean, but changed soil available Cd (ACd) concentrations and caused different effects in the rhizosphere soils of the two crop species. However, there was little difference in bacterial community diversity for the same crop species under the two planting modes. *Proteobacteria, Chloroflexi, Acidobacteria, Actinobacteria* and *Firmicutes* were the dominant phyla in the soybean and corn rhizospheres. In ecological networks of bacterial communities, intercropping soybean (IS) had more module hubs and connectors, whereas intercropped corn (IC) had fewer module hubs and connectors than those of corresponding monoculture crops. Soil organic matter (SOM) was the key factor affecting soybean rhizosphere bacterial communities, whereas available nutrients (N, P, K) were the key factors affecting those in corn rhizosphere. During the cropping season, the concentration of soil available phosphorus (AP) in the intercropped soybean–corn was significantly higher than that in corresponding monocultures. In addition, the soil available potassium (AK) concentration was higher in intercropped soybean than that in monocropped soybean.

**Conclusions:**

Intercropped soybean–corn lead to an increase in the AP concentration during the growing season, and although crop absorption of Cd was not affected in the Cd-contaminated soil, soil ACd concentration was affected. Intercropped soybean–corn also affected the soil physicochemical properties and rhizosphere bacterial community structure. Thus, intercropped soybean–corn was a key factor in determining changes in microbial community composition and networks. These results provide a basic ecological framework for soil microbial function in Cd-contaminated soil.

**Supplementary Information:**

The online version contains supplementary material available at 10.1186/s12866-022-02468-3.

## Background

Long-term application of phosphate fertilizers, pesticides, sewage irrigation and municipal waste has led to cadmium (Cd) to becoming a major pollutant in agricultural soils [1, 2]. Soil Cd pollution causes serious effect of agricultural safety and human health [3–5], because Cd is toxic to biological organisms [6]. Soil microorganisms have important roles in transforming soil organic matter and increasing soil nutrient availability and thus affect the growth, development, yield and quality of crops [7]. Microbes also affect soil nutrient supplies by producing various organic acids, hormones, antibiotics, alcohols, vitamins and other compounds [8, 9]. Species, numbers and activities of soil microorganisms can be reliable biological indicators of soil nutrient conditions [8, 10]. Therefore, it is urgent to understand the influencing factors of rhizosphere soil microorganisms in cadmium contaminated soil.

Intercropping is a high-efficiency planting pattern of planting in which two or more crop species are planted simultaneously in the same field [11, 12]. Appropriate intercropping can regulate absorption of Cd and other heavy metals; inhibit diseases, insects and weeds; promote efficient crop use of soil nutrients; improve crop yield and land use efficiency; and facilitate the development of sustainable agricultural production [13, 14]. Intercropping has become a new strategy for efficient and safe use of heavy metal-contaminated soils in recent years. It can moderate negative effects of heavy metals on soil microorganisms by regulating soil physicochemical properties, soil microbial diversity and plant root exudates [15, 16]. For example, tree–herb intercropping can alleviate toxic effects of heavy metals on soil microbes and alter their composition in contaminated soils [17, 18]. Maize–tomato intercropping has been shown to reduce pH levels and urease activity, increases acid phosphatase activity, and changes root microorganism population structure in Cd contaminated soils [19].

Soybean–corn intercropping is widely practised by farmers in China. Previous studies on intercropping have focused primarily on crop yields, use of heat and light resources and nutrient uptake [20–23]. Although intercropping can affect soil bacterial community diversity and improve soil nutrient management [24], little is known about the interactions between plant heavy metal absorption by crops, soil properties, and soil bacterial communities in soybean–corn intercropping systems on metal(loid)-contaminated soils. Therefore, the aim of this study was to investigate changes in soil properties and bacterial communities in Cd-contaminated soil under soybean–corn intercropping by sampling over time. The objectives were to determine whether different planting patterns affected soil properties and bacterial communities, and more importantly, whether changes in soil properties and bacterial communities moderated the negative effects of Cd.

## Results

### Concentrations of available cadmium in the soil and cadmium in plant tissues

**Fig. 1:**
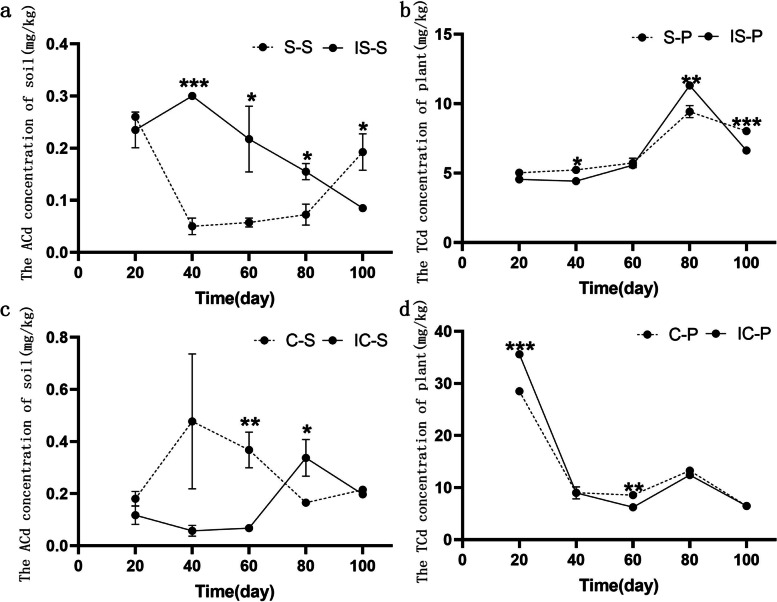
The concentration of ACd of intercropping and monoculture crop soil and TCd of crop intercropping and monoculture crop at five time points. **a** ACd concentration of intercropping and monoculture soybean soil; **b** TCd concentration of intercropping and monoculture soybean plant; **c** ACd concentration of intercropping and monoculture corn soil; **d** TCd concentration of intercropping and monoculture corn plant. S-S: monoculture soybean soil, IS-S: intercropping soybean soil, C-S: monoculture corn soil, IC-S: intercropping corn soil, S-P: monoculture soybean plant, IS-P: intercropping soybean plant, C-P: monoculture corn plant, IC-P: intercropping corn plant

In the soybean monoculture, the available Cd (ACd) concentration in the rhizosphere soils decreased and then increased, with the lowest value occurring on day 40. However, in intercropped soybean, the opposite trend was observed (Fig. 1a). The concentration of ACd was significantly (*P* < 0.05) different between intercropped and monoculture soybean rhizosphere soils at 40, 60, 80, and 100 days (Fig. 1a). The ACd concentration in the monocropped corn rhizosphere soil all decreased from day 20 and 60, then increased on day 60 and 80, and finally decreased from day 80 to 100, with the lowest value on day 40. However, the opposite trend was observed in intercropped corn soil (Fig. 1c).

The dynamic changes of Cd concentration were similar in intercropped and monocropped soybean/corn plants throughout the crop planting season (Fig. 1b, d). Cd concentration in intercropped and monocropped soybean gradually increased from day 20 to 80, reached the highest on day 80, and then decreased from day 80 to 100 (Fig. 1b). In intercropped and monocropped corn, Cd concentration gradually decreased from day 20 to 40, and then stabilized beginning on day 40 (Fig. 1d).

### Effects of soybean and corn intercropping on soil physicochemical properties

**Fig. 2:**
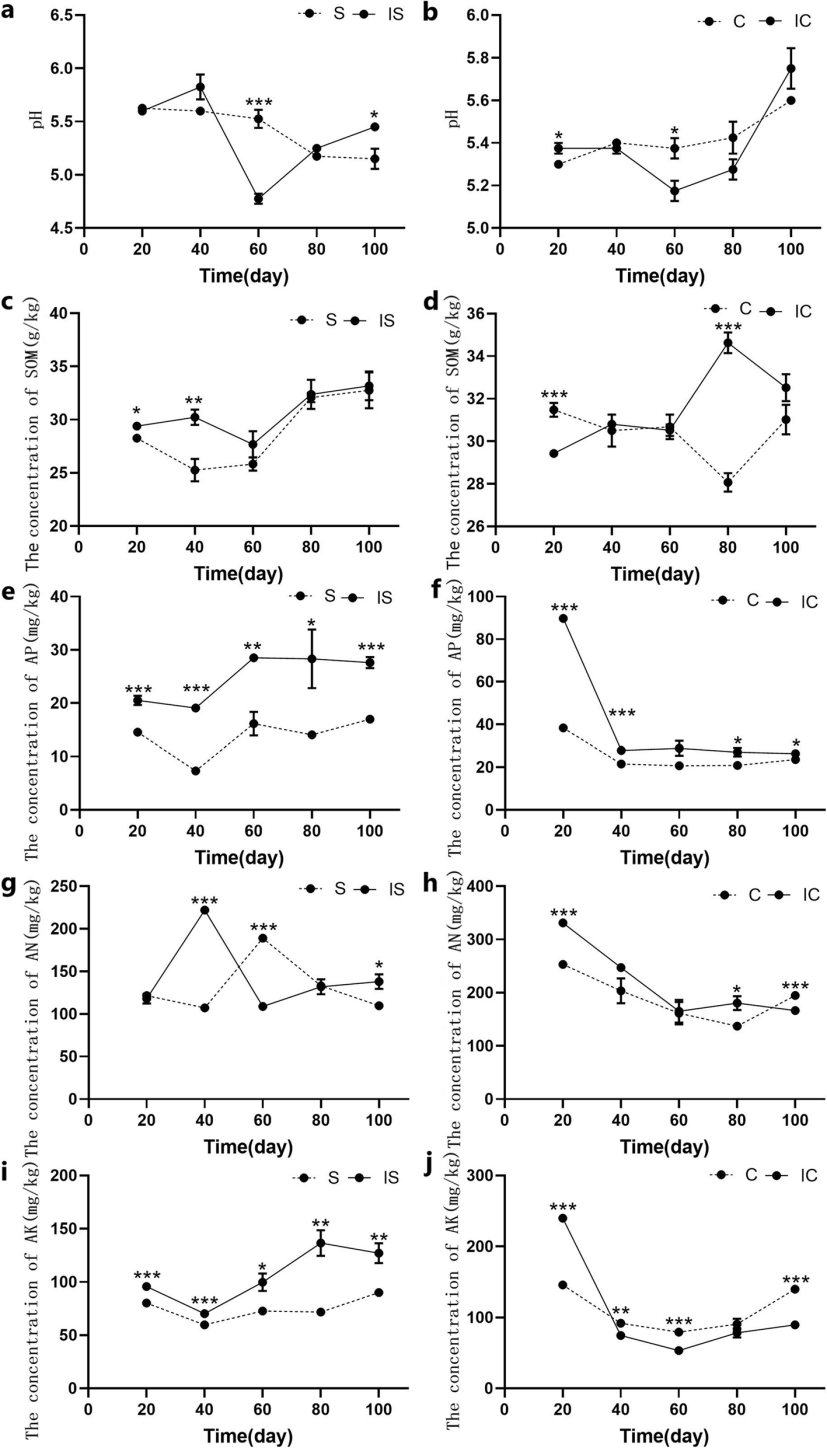
The soil physicochemical properties of soybean and corn intercropping soil. **a** pH value of intercropping and monoculture soybean soil; **b** pH value of intercropping and monoculture corn soil; **c** SOM concentration of intercropping and monoculture soybean soil; **d** SOM concentration of intercropping and monoculture corn soil; **e** AP concentration of intercropping and monoculture soybean soil; **f** AP concentration of intercropping and monoculture corn soil; **g** AN concentration intercropping and monoculture soybean soil; **h** AN concentration of intercropping and monoculture corn soil; **i** AK concentration of intercropping and monoculture soybean soil; **j** AK concentration of intercropping and monoculture corn soil.S: monoculture soybean, IS: intercropping soybean, C: monoculture corn, IC: intercropping corn. SOM: soil organic matter, AP: available phosphorus, AN: available nitrogen, AK: available kalium

Soil properties were measured in samples collected 20, 40, 60, 80, and 100 days after sowing (Fig. 2). Physicochemical properties were compared between monocropped and intercropped corn and soybean at each sample point (Fig. 2). Rhizosphere soil pH decreased in monocropped soybean, but increased in corn (Fig. 2a, b). In addition, rhizosphere soil pH in intercropped soybean and corn decreased first but then increased, with the lowest values occurring on day 60 (Fig. 2a, b). During the soybean season, the concentrations of available phosphorus (AP) and available potassium (AK) were significantly higher in intercropped soybean soil than in the monocropped soybean soil (Fig. 2e, i). During the corn season, the soil AP concentration was significantly higher in intercropped corn than in monocropped corn (Fig. 2f). The concentration of soil organic matter (SOM) of intercropping corn soil was lower in intercropped corn than in monocropped corn on day 20, although it was higher at other times (Fig. 2d). The AK concentration was higher in intercropped corn soil than in monocropped corn soil on day 20, although it was lower than that at other time (Fig. 2j). The available nitrogen (AN) concentration was lower in intercropped corn than in the monocropped corn on day 100, although it was higher than that at other time points (Fig. 2h).

### Effects of soybean and corn intercropping on the α and β diversity of bacterial communities in the crop rhizosphere soil

**Fig. 3:**
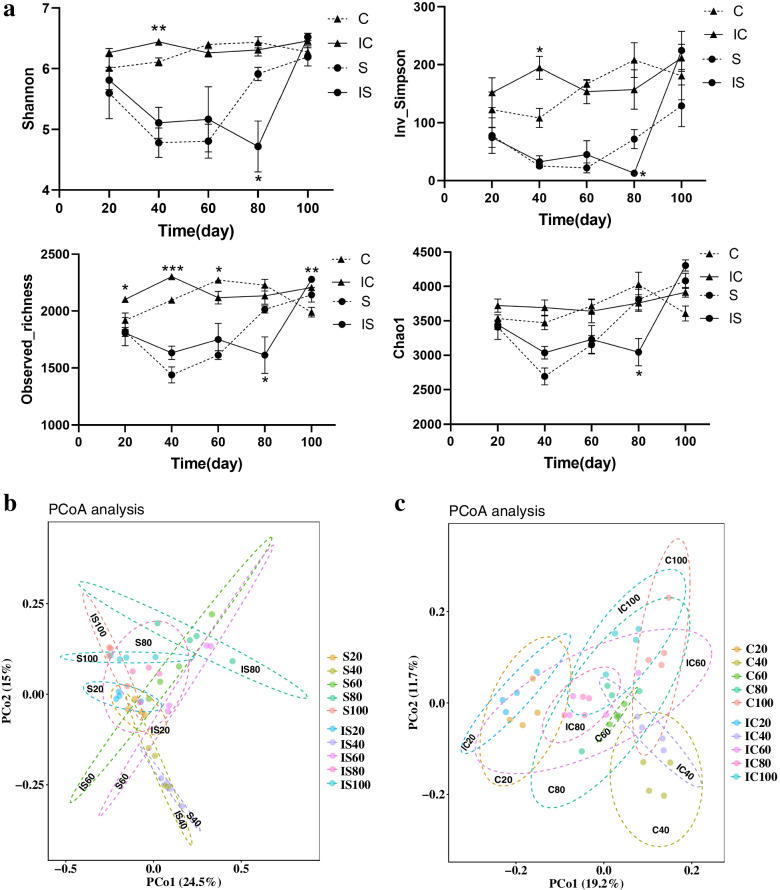
The α(**a**) and β(**b**, **c**) diversity in crop rhizosphere soil. **a** α diversity of monoculture and intercropping soybean/corn rhizosphere soil; **b** principal co-ordinates analysis (pCoA) analysis of the monoculture and intercropping soybean rhizosphere soil; **c** principal co-ordinates analysis (pCoA) analysis of the monoculture and intercropping corn rhizosphere soil. S: monoculture soybean, IS: intercropping soybean, C: monoculture corn, IC: intercropping corn

A total of 3,881,770 high-quality sequences were obtained from Illumina MiSeq sequencing of 80 rhizosphere soil samples. The observed richness operational taxonomic unit (OTU) numbers and Shannon and inverse Simpson indices decreased first but then increased with time in monoculture and intercropped soybean rhizosphere soil. In the monocropped and intercropped corn rhizosphere soils, there were no significant differences in richness and diversity at different times (Fig. 3a). The Chao1 estimated richness of the monocropped and intercropped corn and soybean rhizosphere soil gradually increased through the five sampling time points (Fig. 3a). According to Student’s *t* test results, the α diversity indices were significantly different between the monocropped corn and soybean on days 40, 60 and 80 as well as the intercropped corn and soybean on days 20, 40 and 80 (Fig. 3a). Significant differences were also detected in α diversity indices between the monocropped and intercropping soybean on day 80 and between the monocropped and intercropped corn on day 40 day (Fig. 3a). The α diversity of intercropped/monocropped corn was higher than that of intercropped/monocropped soybean. In general, there was a little difference in bacterial community α diversity across rhizosphere soils of the same crop sampled at different times.

The principal coordinate analysis (PCoA) (Fig. 3b, c) and PERMANOVA results (Tables S1 and S2) showed that the rhizosphere bacterial community structure of monocropped soybean was significantly different from day 20 to 80, whereas that of intercropped soybean was significantly different on day 20, 40, 60(80) and 100. In the monocropped and intercropped corn, rhizosphere bacterial community structure was significantly different among the five stages (*P* < 0.05). Rhizosphere bacterial community structures was significantly different in the monocropped corn and soybean among five stages and in intercropped soybean and corn on days 20(40), 60, 80 and 100. In the monocropped and intercropped soybean, rhizosphere bacterial community structure was significantly different on days 20, 40 and 80. In monoculture and intercropped corn, rhizosphere bacterial community structure was significantly different on day 40 and 60.

### Molecular ecological networks of bacterial communities under different planting modes

In our study, only a few microbes had many connections with others according to the phylogenetic molecular ecological network (pMENs) results (Figs. S1, S2). The group IS had the higher average connectivity (avgK:5.958) than S group. But the group IC had the lower average connectivity (avgK:2.2) than C group. The nodes and links for all four groups including S (537, 1380), IS (574, 1710), C (581, 918), and IC (500,550) were obtained in this study (Table 1). The values of average clustering coefficient (avgCC), average geodesic distance (GD) and modularity of empirical networks were significantly higher than those of their corresponding random values with identical sizes in our study (Table 1). In our study, 1.82% of bacterial OTUs were module hubs and 1.14% of OTUs that were connectors. The group IS had the more module hubs and connectors than corresponding monoculture crop group did, while the group IC had the fewer module hubs and connectors (Tables S3, S4).

**Table 1:** The properties of the empirical and random networks under different cultivation mode. S: monoculture soybean, IS: intercropping soybean, C: monoculture corn, IC: intercropping corn

Groups	Empirical Network						Random networks(100)		
	RMT threshold	nodes	links	Average degree (avgK)	Average clustering coefficient (avgCC)	Modularity (fast_greedy)	Average clustering coefficient (avgCC)	Average path distance (GD)	Modularity (fast_greedy)
**S**	0.8	537	1380	5.14	0.157	0.524	0.046 +/− 0.005	3.581 +/− 0.034	0.398 +/− 0.005
**IS**	0.8	574	1710	5.958	0.185	0.562	0.051 +/− 0.005	3.445 +/− 0.032	0.358 +/− 0.004
**C**	0.8	581	918	3.16	0.123	0.793	0.011 +/− 0.003	4.549 +/− 0.054	0.600 +/− 0.006
**IC**	0.8	500	550	2.2	0.058	0.814	0.005 +/− 0.003	5.620 +/− 0.157	0.772 +/− 0.007

### Changes in bacterial taxa in rhizosphere soils at different times

**Fig. 4:**
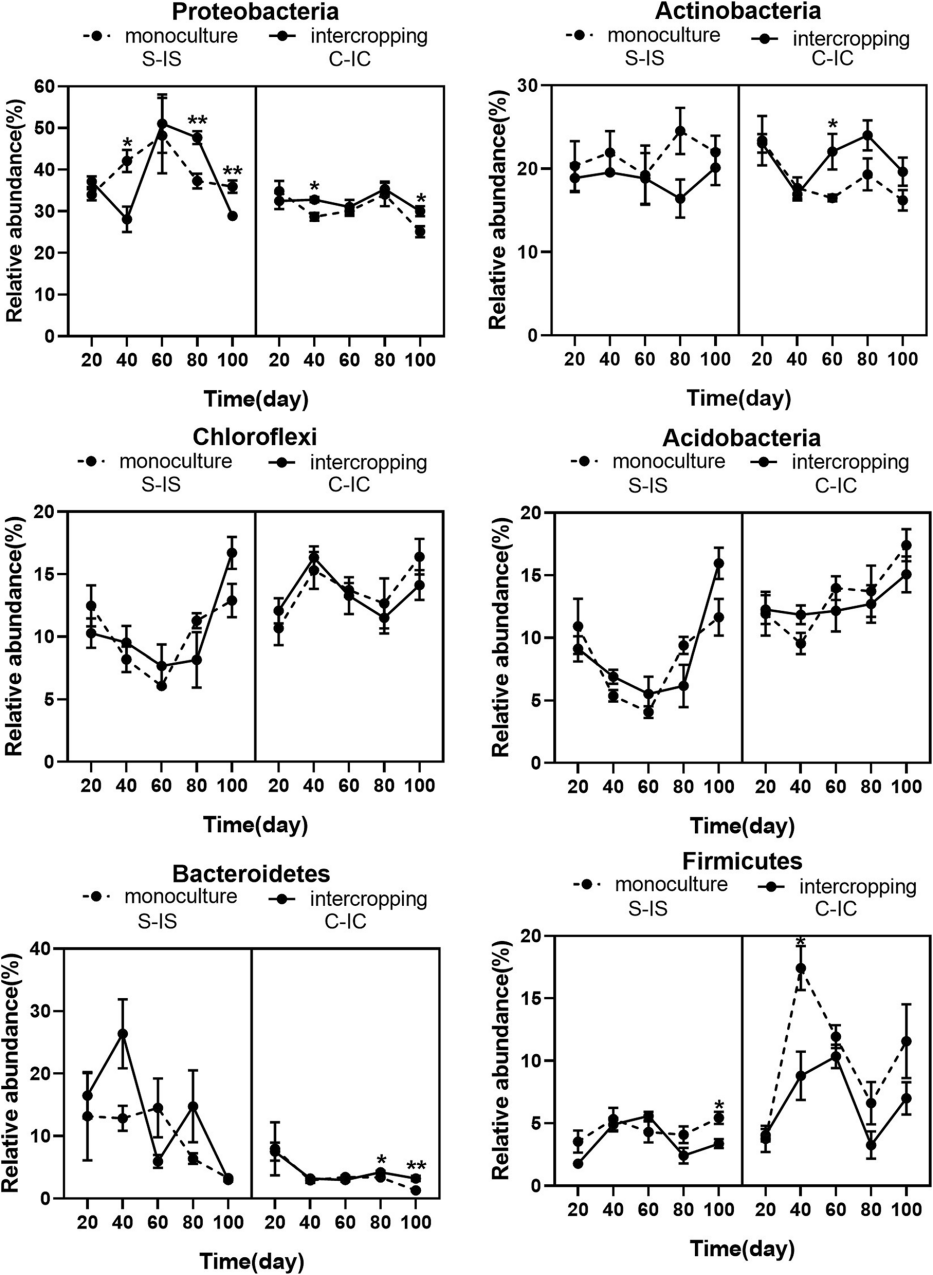
The main phyla intercropping and monoculture crop rhizosphere soil samples. S-IS: monoculture and intercropping soybean, C-IC: monoculture and intercropping corn

The soil bacterial communities changed significantly at different growth stages. The phyla *Actinobacteria, Proteobacteria, Chloroflexi, Firmicutes, Acidobacteria* and *Bacteroidetes* accounted for 88.02 and 96.38% of rhizosphere soil communities in soil samples (Fig. S3). The main phyla were displayed in Fig. 4. The relative abundance of *Proteobacteria* in intercropped soybean rhizosphere soil was significantly lower than that in the monocropped soybean rhizosphere soil at days 40 and 100, but was significantly higher on day 80. The relative abundance of *Proteobacteria* in intercropped corn rhizosphere soil was significantly higher than that in the monocropped corn rhizosphere soil on days 40 and 100. There were no significant differences in relative abundance of *Actinobacteria, Chloroflexi* and *Acidoacteria* between the intercropped and monocropped crop rhizosphere soils. However, a significant difference in the main phyla was observed between soybean and corn rhizosphere soil. The relative abundance of *Bacteroidetes* in intercropped corn rhizosphere soil was significantly higher at days 80 and 100, but the relative abundance of *Firmicutes* in the intercropped soybean rhizosphere soil was significantly lower on day 100. Compared with that of the other phyla, the abundance of *Firmicutes* was also lower in the intercropped corn than in the corresponding monocropped corn on day 40.

### Analysis of correlations analysis between bacterial communities and environmental factors

The Pearson correlation analysis results showed that only SOM was positively correlated with bacterial community structure in the rhizospheres of intercropped and monocropped soybean (*P* < 0.01). However, there were no significant correlations between rhizosphere soil bacterial α diversity and physicochemical properties (*P* > 0.05) in the intercropped and monocropped corn (Table S5).

**Fig. 5:**
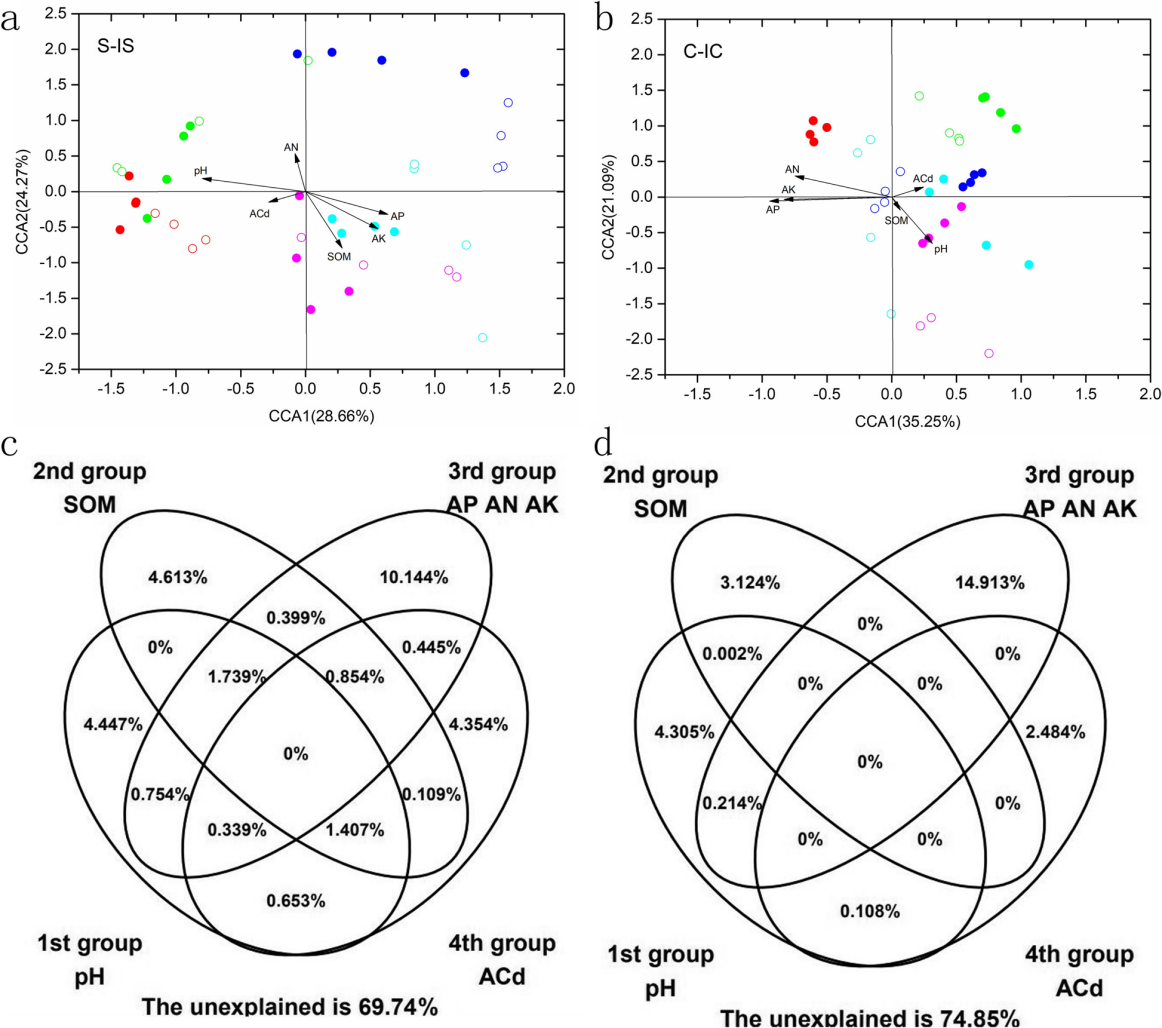
The CCA (**a**, **b**) and CCA-based VPA (**c**, **d**) between intercropping and monoculture crop rhizosphere soil samples. **a** CCA of intercropping and monoculture soybean; **b** CCA of intercropping and monoculture corn; **c** CCA-based VPA of intercropping and monoculture soybean; **d** CCA-based VPA of intercropping and monoculture corn. Solid circle: intercropping soybean/corn; hollow circle: monoculture soybean/corn. Red: 20 day, green: 40 day, blue: 60 day, cyan: 80 day, magenta: 100 day

The mantel test and canonical correspondence analysis (CCA) were performed to assess correlations between physicochemical properties and bacterial community composition (Table 2 and Fig. 5a, b). The results showed that soil organic matter was significantly positively correlated with rhizosphere soil bacterial communities in the monocropped and intercropped soybean and that AP, AK and AN were significantly positively correlated with the rhizosphere soil bacterial communities in monocropped and intercropped corn (*P* < 0.05, Table 2). According to the CCA-based variation partitioning analysis (VPA) results, 4.45, 4.61, 4.35 and 10.14% of the variation in the rhizosphere soil bacterial community in the monocropped and intercropped soybean systems could be respectively explained by the pH; SOM; ACd; and AP, AK, and AN (Fig. 5c). Moreover, their interaction could explain 11.16% of the variation, leaving 69.74% of the variation unexplained (Fig. 5c). In addition, 4.30, 3.12, 2.48 and 14.91% of the variation in the rhizosphere soil bacterial communities in the monocropped and intercropped corn systems could be respectively explained by the pH; SOM; ACd; and AP, AK, and AN (Fig. 5d). Their interaction could explain 0.34% of the variation, leaving 74.85% of the variation unexplained (Fig. 5d).

**Table 2:** The mantel analysis based on Bray_curtis distance under different cultivation modes. S: monoculture soybean, IS: intercropping soybean, C: monoculture corn, IC: intercropping corn. SOM: soil organic matter, AP: available phosphorus, AN: available nitrogen, AK: available kalium. S_IS: monoculture and intercropping soybean, C_IC: monoculture and intercropping corn

Factors	S_IS		C_IC	
	r	p	r	p
**pH**	0.0467	0.287	0.138	0.072
**SOM**	0.1405	*	0.0374	0.339
**AP**	0.0813	0.134	0.2838	*
**AN**	0.0578	0.233	0.2276	**
**AK**	0.1098	0.107	0.3041	**
**ACd**	0.0827	*	−0.0378	0.607

## Discussion

The effects of intercropping on Cd accumulation in plants were different. In our study, the dynamic trends of plant Cd were consistent between the intercropping and monocropping systems throughout growing season. The cadmium concentration of corn straw gradually decreased with increasing time after sowing, whereas the cadmium concentration of soybean straw first gradually increased and then decreased with increasing time after sowing. These results strongly agree with the previous findings that Cd concentration of plants was not significantly affected in grape and *Solanum nigrum* L. [25]. Furthermore, another study found that the Cd concentration of shoot cells in intercropped *V. faba* shoot cells was greater than that of monocropped shoot cells [26]. These results may be due to the plant species used, change in plant rhizosphere environments due to intercropping and effects on plant absorption of heavy metals [26, 27].

Compared with monocropping, intercropping can change soil nutrient composition [28]. In this study, the AP and SOM concentrations were higher in the IC and IS groups than in their corresponding monocultures, which is consistent with the previous observations [24, 29, 30]. The concentration of AP was significantly higher in the intercropping system than in the monocultures. Alternatively, this phenomenon may have resulted from greater nitrogen fixation, which can acidify the rhizosphere soil of legumes and activate the organophosphorus in the soil, compared to that from the monoculture [31]. The soil AK concentration in the IS samples was also higher than that in the corresponding monoculture samples. This results might be explained by differences in nutrient use, given that intercropping affected the soil nutrient pool [32]. The AN concentration in soil under corn gradually decreased, whereas that in soybean gradually increased and then decreased. In addition, soil AN concentration were higher under intercropped corn than under monoculture corn except on day 100. These results were likely due to nitrogen fixation by soybean and improved supply of nitrogen to intercropped corn and soybean, with corn undergoing increased N uptake [33, 34].

Slight acidification in the rhizosphere was observed with the growth of soybean, which is consistent with results typically observed in common bean [35]. Changes in rhizosphere soil pH depend on cation-anion balance, which is greatly affected by nitrogen nutrition [36]. In this experiment, the rhizosphere pH in IS and S treatment was different. The rhizosphere pH in IS was significantly lower than that in the monoculture on day 60. It may have resulted from an increase in proton release by the intercropped soybean due to greater N_2_ fixation compared to the sole crop, as a consequence of acidification is expected to occur when the amount of proton released when the intensity of N_2_ fixation by soybean [37]. By contrast, rhizosphere pH in IS was significantly higher than that in the monocropped on day 100. This result might be explained by lower N_2_ fixation that occurred in intercropped soybean than in the monoculture with crop growth. In the rhizosphere of corn, alkalization was observed in the monocropped or intercropped with corn intercropping systems, which is in line with the previous results [24, 29]. The rhizosphere pH of the IC was significantly lower than that in the monocropped on day 60. This result might be explained by interspecific competition for the uptake of inorganic N forms [38], with intercropped corn being more competitive than the monocropped corn in accessing nitrate. Rhizosphere soil pH in IC and IS was higher than that in corresponding monocultures at 100d. The pH of all the samples from the soybean soil all decreased, while that of the corn soil increased, but no significant acidification was observed in rhizosphere soil. Fu et al. [24] and Betencourt et al. [29] reported similar results.

The rhizosphere soil ACd increased first and then decreased in IS, whereas the pattern was the opposite in the corresponding monoculture. The changes in corn rhizosphere soil ACd were opposite to those in soybean. The concentrations of ACd in IS and IC groups were significantly different from those in corresponding monoculture from day 40, and the differences of ACd concentrations between the monocultures and intercropping systems gradually decreased with cultivation time, which may be due to the roots of the intercropped plants secreting numerous organic acids and consequently altering the mobility of soil Cd in the intercropping systems [39, 40]. However, there were significant differences in ACd concentration between IS and S, which indicated that the soybean activated the soil Cd [41] . Consequently, the results showed that the soybean–corn intercropping system altered the cadmium concentrations in cadmium-polluted soil.

Soil microorganisms participate in many ecological processes and greatly influence soil quality and function [42]. The dominant phyla in soybean and corn rhizosphere soil were *Proteobacteria, Chloroflexi, Acidobacteria, Actinobacteria* and *Firmicutes*, which is similar to results reported in previous studies [24]. The main phyla were similar in rhizosphere of the same crop in monoculture and intercropping systems, but there were slight differences at all growth stages. The significant differences in the main phyla were observed between soybean and corn rhizosphere soil, likely because of the crop species genotype [43, 44]. Comparing the bacterial community diversity of soybean and corn under the two planting modes, we also found that the same crop showed little difference under the two planting modes [45]. However, the bacterial community diversity was significantly higher in corn rhizosphere soil than in the soybean rhizosphere soil. Therefore, compared with that of the soybean soil, the bacterial diversity of the maize soil was enriched, which might have affected the absorption of ACd by corn.

The network interactions within the rhizosphere bacterial communities of the four different groups were also analysed. Owing to their relatively strong robustness and consistency, networks based on random matrix theory could accurately reflect various complex biological systems because of relatively strong robustness and consistency [46]. In general, a more complex network indicates a more stable community structure [47, 48]. In this study, the modularity values for all the groups were higher than those of corresponding randomized networks (Table 1), which indicated all pMENs appear to be modular [49]. Interspecies interaction was significantly different between intercropped and monoculture crop species. There were significantly more bacterial network nodes and links in the IS group compared with the corresponding monoculture crop group but significantly fewer in the IC group (Table 1). Therefore, the network within intercropped soybean was more complex than that in the monoculture, whereas the network within intercropped corn was simpler than that in the monoculture. Thus, soybean–corn intercropping affected the co-occurrence network patterns of the rhizosphere bacterial communities.

There were large changes among these module hubs and connectors between the intercropped and monocropped soybean and corn networks (Table S3). These changes may have occurred because of ubiquitous taxa with specific functions. These shared species of different soils take apart in plant-microbe interactions through their functions [50, 51], while keystone species are generally crucial for determining ecological functions and ecosystem stability of an entire ecological network [52, 53]. However, we still lacked a comprehensive understanding of these shared and key species. Therefore, further research is needed to provide direct evidence for network analysis of complex microbial communities.

In our study, we found a slight difference in bacterial diversity, but the bacterial communities of intercropped and corresponding monocropped soybean were significantly different at 20, 40 and 80d, while those of maize were significantly different at the 40 and 60d. This result indicated that cultivation time is also an important factor affecting rhizosphere bacterial community except for crop the planting pattern [49]. While these rhizosphere microorganisms also regulate the rhizosphere soil environment through plant-microbe interactions [50]. It also will be our next research focus that regulating the cadmium concentration in cadmium-contaminated soil through plant-microbial interactions.

## Conclusion

Compared with monocropping, intercropping led to increased concentrations of AP and SOM in rhizosphere soils of soybean and corn compared with those in corresponding monoculture. Changes in soil nutrients due to intercropping were determined to be important factors in determining the structure and diversity of bacterial communities. Soil organic matter and ACd were significantly correlated with bacterial communities of soybean, whereas AP, AN, and AK were key factors affecting communities in corn rhizosphere soils. The findings suggested that intercropping increased stability of soybean rhizosphere community structure but decreased the stability of the corn rhizosphere community structure. However, further research is still needed to provide direct evidence for network analyses on complex microbial communities in a cooperative environment. These results provide important insights into how soil ACd, soil nutrients, and bacterial diversity are affected via plant–microbe interactions in legume–cereal intercropping agroecosystems in Cd-contaminated soils.

## Materials and methods

### Site description, experimental design and sample collection

The experiment was conducted in Zhuzhou City (27°43′25.6″N, 113°8′3.8″E), Hunan Province, China. The region has a subtropical monsoon humid climate, the average annual temperature is 17.5 °C–18 °C, the average rainfall amount is 1400–1500 mm, and the average annual sunshine is 1500 h–1600 h. The field experiment included two monocropping systems, monocropped corn (C) and monoculture soybean (S), and soybean-corn intercropping system (ICS) in a typical cadmium-polluted fallow zone. The plants used were compact and high-cadmium accumulating corn (Denghai 605) and a major soybean cultivars (Xiangchundou 26) grown in Hunan Province. Corn and soybean were sown on April 6, 2019, and harvested on July 30, 2019. The soil chemical properties in the top 20 cm were as follows: pH 5.51, total Cd 1.98 mg∙kg^− 1^, available Cd 1.16 mg∙kg^− 1^, total N 1.99 g∙kg^− 1^, organic matter 31.3 g∙kg^− 1^; total P, 0.626 g∙kg^− 1^; total K, 11.1 g∙kg^− 1^; alkali hydrolysable N, 112 mg∙kg^− 1^; Olsen-P, 12.6 mg∙kg^− 1^; and exchangeable K, 64 mg∙kg^− 1^.

**Fig. 6:**
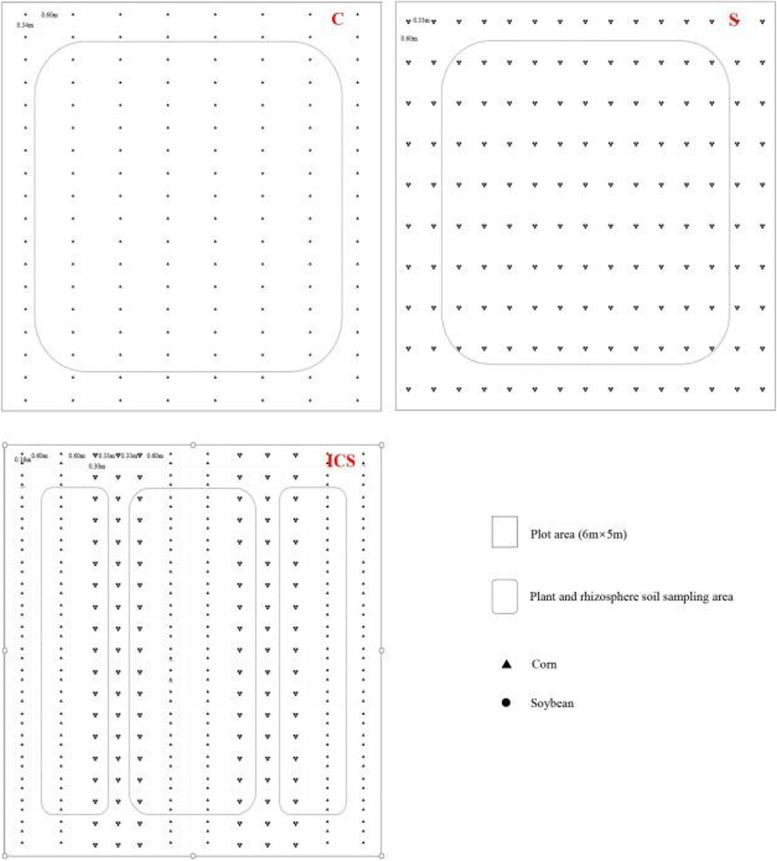
Diagram of different planting patterns. S: monoculture soybean; C: monoculture corn; ICS: intercropping corn and soybean

In the soybean-corn intercropping system, the ratio of corn rows to soybean rows was 2:3. In the monoculture system, the row spacings of the corn and soybean plants were 60 cm and 33 cm, respectively. The plant spacings of the corn plants and soybean plants were 32 cm and 60 cm, respectively. The distance between corn and soybean rows was 60 cm (Fig. 6). The planting density for corn was 52,500 hm^− 2^, and for soybean was 150,000 hm^− 2^. The intercropping systems were planted at same density, but the plant spacings of corn and soybean plants were 16 cm and 30 cm, respectively. The plot size was 30 m^2^, and each treatment was applied in four replicate plots. The nitrogen fertilization for corn was divided into two parts, 112.5 kg∙hm^− 2^ for base fertilizer and 105.75 kg∙hm^− 2^ for topdressing. The phosphorus and potassium fertilization were used as base fertilizer at 112.5 kg∙hm^− 2^ and 112.5 kg∙hm^− 2^ for corn. The nitrogen, phosphorus and potassium fertilization were used as base fertilizer 67.5 kg∙hm^− 2^, 67.5 kg∙hm^− 2^ and 67.5 kg∙hm^− 2^ for soybean. In IMS, the nitrogen topdressing for IM applied with a distance of 40 cm from the maize rows to the soybean rows. There was no irrigation applied during crop growth.

Plant and rhizosphere soil samples were collected at 20, 40, 60, 80 and 100 days after sowing. In each plot, samples were randomly collected from five points and mixed into one composite sample. The plant samples were separated into straw (stems and leaves) and grain, which were then weighed after oven-drying 80 °C until a constant weight was achieved. The rhizosphere soil was collected by shaking plant roots. The rhizosphere soil samples were divided into two parts. One part was used for DNA extraction, and the other part was air-dried and used for analyze soil property analysis.

### Determination of plant and soil physiochemical properties

The soil pH was measured via potentiometry with a pH meter (PB-10, Sartorious, Germany). Soil available cadmium (ACd) was extracted with calcium chloride [54]. Plant samples were digested in a mixture of HNO_3_ and HClO_4._ The concentration of ACd and Cd were measured with graphite furnace atomic absorption spectrometry (ZEEnit 700 P, Analytik Jena AG, Germany). The concentrations of soil organic matter (SOM) and available nitrogen (AN) were measured using volumetric methods [55, 56]. The concentration of available phosphorus (AP) was measured by UV-Vis spectrophotometry [56]. Available potassium (AK) was determined by the inductively coupled plasma-atomic emission spectrometry (ICP-AES).

### DNA extraction, PCR amplification and pyrosequencing

In total, 80 rhizosphere soil samples were collected and sequenced. The soil DNA was extracted using a Fast DNA Spin Kit for soil (MP Biomedicals LLC, USA) according to the manufacturer’s instructions. DNA was stored at − 20 °C before use. The V3-V4 region of the 16S rRNA gene was amplified with primer pair 338F (5′-ACTCCTACGGGAGGCAGCAG-3′) and 806R (5′-GGACTACHVGGGTWTCTAAT-3′) [57]. Both the forward and reverse primers were tagged with unique barcodes to distinguish different samples. PCR and sequencing were performed at Majorbio Bio-Pharm Technology Co. Ltd., Shanghai, China. Sequencing was performed on an Illumina MiSeq platform using a PE250 kit. The sequencing data were deposited in the NCBI Sequence Read Archive database under BioProject ID PRJNA662201.

### Data and statistical analyses

Data analysis was performed using the open, web-based platform, Galaxy (http://mem.rcees.ac.cn:8080) [58]. Briefly, 12 bp barcode sequences were used to separate the different soil samples. The forward and reverse sequences were then combined with a minimum 30 bp overlap length and a maximum 250 bp maximum overlap length using the FLASH program [59]. Combined sequences with low quality were removed. Subsequently, the reads were clustered into operational taxonomic units (OTUs) with 97% similarity using UPARSE [60]. The OTU table was resampled with 17,093 sequences.

The α-diversity indices and the relative abundances of phyla and genera were calculated in this study. Differences in microbial community structure were investigated by weighted principal coordinate analysis (PCoA) based on weighted UniFrac matrix and dissimilarity tests (nonparametric permutational multivariate analysis of variance (PERMANOVA) test based on Bray Curtis) [61, 62]. The Mantel test was used to evaluate the correlations between physicochemical properties and the microbial communities. The canonical correspondence analysis (CCA) and CCA-based variation partitioning analysis (VPA) were further performed to determine the relative contribution of environmental variables to the bacterial community. To evaluate the differences in soil physicochemical properties between the intercropped and corresponding monocropped soybean and corn samples during various stages, Student’s *t* tests were performed in the Excel 2017.

### The network analysis

Phylogenetic molecular ecological networks (pMENs) of the four treatment groups (S, IS, C and IC) were constructed based on Spearman rank correlation matrices using the molecular ecological network analysis pipeline (MENA, http://ieg4.rccc.ou.edu/mena/login.cgi) [46, 63, 64]. The process was followed as described by Deng [64], and the correlations only above a specific threshold (0.80) were used to calculating the network eigenvalues. The network plots were visualized with the software Cytoscape 3.6.0.

## Supplementary Information

**Additional file 1.**

**Additional file 2.**

**Additional file 3.**

**Additional file 4.**

**Additional file 5.**

**Additional file 6.**

## Data Availability

All study data are included in the manuscript and its additional files. The sequencing data were deposited in the NCBI Sequence Read Archive database under BioProject ID PRJNA662201.

## Abbreviations

SOM: Soil organic matter
AP: Available phosphorus
ACd: Available cadmium
AN: Available nitrogen
AK: Available potassium
pCoA: The principal coordinate analysis
ADONIS: Nonparametric parametric permutational multivariate analysis of variance of the Adonis function
OTU: Operational taxonomic unit
PERMANOVA: Nonparametric permutational multivariate analysis of variance
