# Supplementary material for: Dynamic changes of rhizosphere soil bacterial community and nutrients in cadmium polluted soils with soybean-corn intercropping
Source: BMC Microbiol. 2022 Feb 15;22:57. doi: 10.1186/s12866-022-02468-3 (PMC8845239; doi:10.1186/s12866-022-02468-3)
Supplement: Supplementary file 1 — Additional file 1. [file 12866_2022_2468_MOESM1_ESM.docx]

**Table S1 The** **PERMANOVA** **analysis of monoculture and intercropping soybean/corn bacterial community composition based on Bray-Curtis distance among different groups.** *: P<0.05. S: monoculture soybean soil, IS: intercropping soybean soil, C: monoculture corn soil, IC: intercropping corn soil.

| **Groups** | **S-IS** | **C-IC** |
| --- | --- | --- |
| **20-20** | 1.7723* | 1.1494 |
| **40-40** | 4.8315* | 2.8177* |
| **60-60** | 1.3923 | 1.6551* |
| **80-80** | 3.9009* | 1.4392 |
| **100-100** | 1.5351 | 1.7663 |
