# Supplementary material for: Dynamic changes of rhizosphere soil bacterial community and nutrients in cadmium polluted soils with soybean-corn intercropping
Source: BMC Microbiol. 2022 Feb 15;22:57. doi: 10.1186/s12866-022-02468-3 (PMC8845239; doi:10.1186/s12866-022-02468-3)
Supplement: Supplementary file 2 — Additional file 2. [file 12866_2022_2468_MOESM2_ESM.docx]

**Table S2 The PERMANOVA analysis of monoculture and intercropping soybean/corn bacterial community composition among different time points based on Bray-Curtis distance among different groups.** *: P<0.05. S: monoculture soybean soil, IS: intercropping soybean soil, C: monoculture corn soil, IC: intercropping corn soil.

| **Groups** | **S-S** | **C-C** | **IS-IS** | **IC-IC** |
| --- | --- | --- | --- | --- |
| **20-40** | 7.1697* | 4.8603* | 4.5031* | 7.0709* |
| **20-60** | 3.9342* | 3.2425* | 3.6541* | 3.3417* |
| **20-80** | 2.7382* | 2.8728* | 5.4628* | 3.705* |
| **20-100** | 2.6891* | 4.5397* | 4.7356* | 4.4791* |
| **40-60** | 4.4768* | 3.3808* | 4.0019* | 3.5203* |
| **40-80** | 6.4767* | 3.7794* | 5.9802* | 4.0533* |
| **40-100** | 7.384* | 4.6432* | 8.0886* | 3.6636* |
| **60-80** | 2.8991* | 1.5553* | 2.0135 | 2.2754* |
| **60-100** | 3.5664* | 3.3594* | 5.0089* | 2.3448* |
| **80-100** | 1.7327- | 3.0059* | 6.9886* | 2.9811* |
