# Supplementary material for: Dynamic changes of rhizosphere soil bacterial community and nutrients in cadmium polluted soils with soybean-corn intercropping
Source: BMC Microbiol. 2022 Feb 15;22:57. doi: 10.1186/s12866-022-02468-3 (PMC8845239; doi:10.1186/s12866-022-02468-3)
Supplement: Supplementary file 3 — Additional file 3. [file 12866_2022_2468_MOESM3_ESM.docx]

**Supplementary figures:**
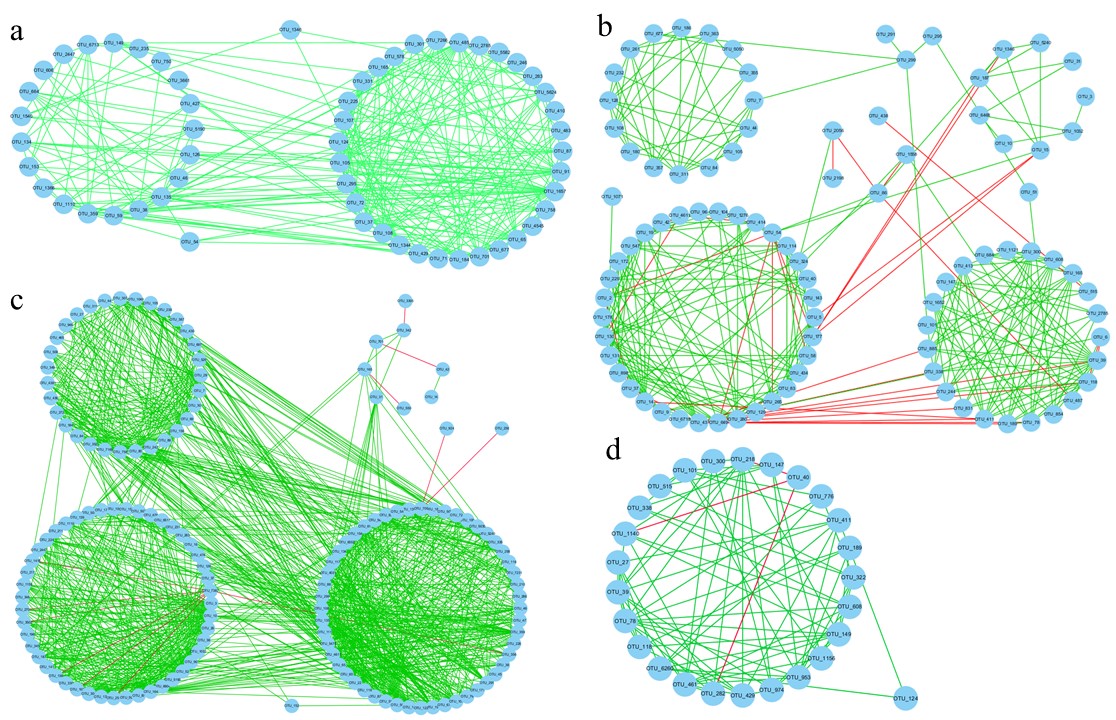


**Figure S1 Phylogenetic molecular ecological networks (pMENs) of bacterial communities under the two planting modes.** Modules with >5 nodes were obtained for bacterial groups, respectively. The links between two nodes show the correlation (red: positive, blue: negative).


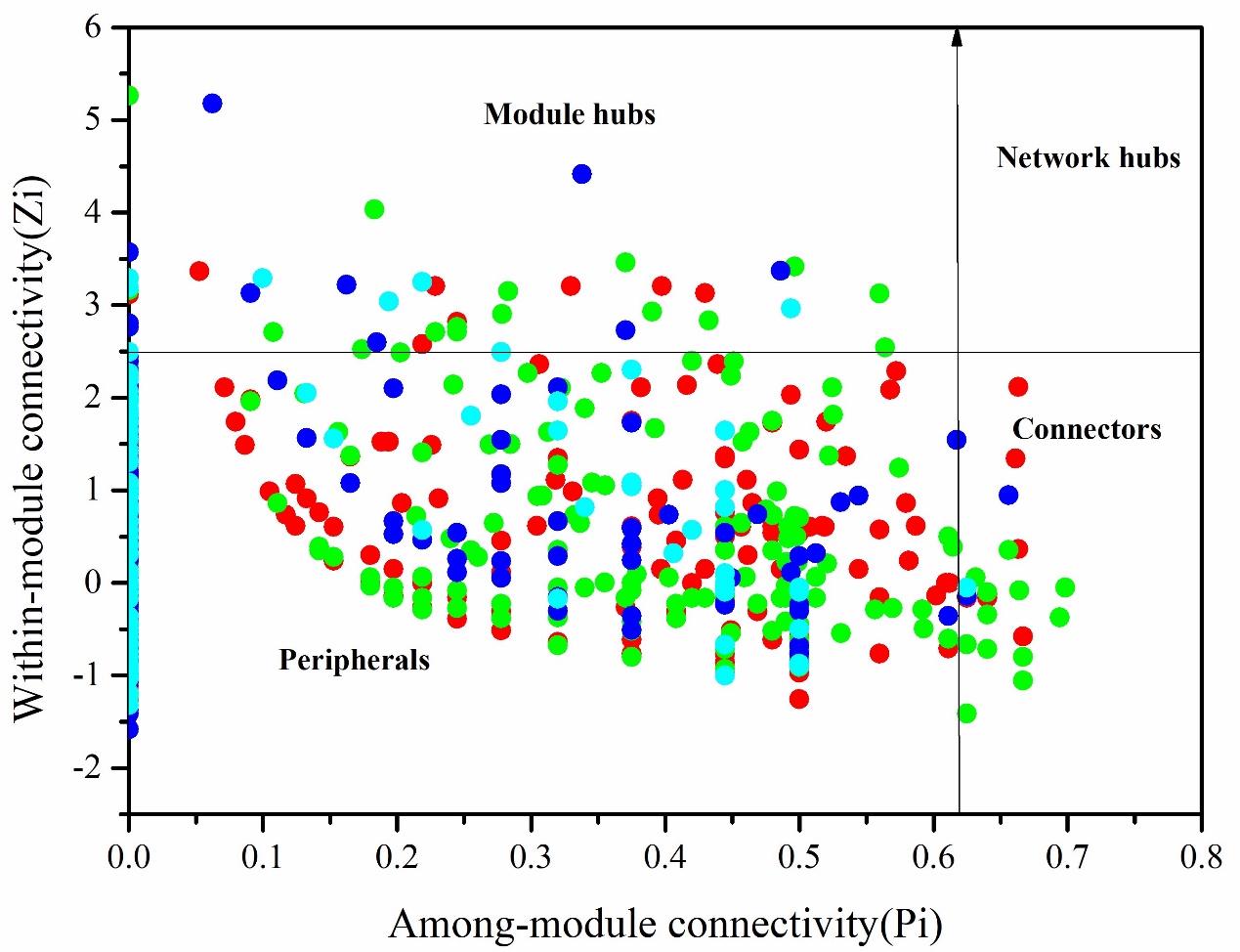

**Figure S2 Summary of module hubs and connectors of the bacterial and fungal (B) communities under the two planting modes.** The OTUs were peripherals whose links mainly stayed within their respective modules. Generalists including module hubs (nodes that highly connected with nodes within their modules, Zi > 2.5) and connectors (nodes that connected with several modules, Pi > 0.62). Red circle: monoculture soybean, green circle: intercropping soybean, blue circle: monoculture corn, cyan circle: intercropping corn.


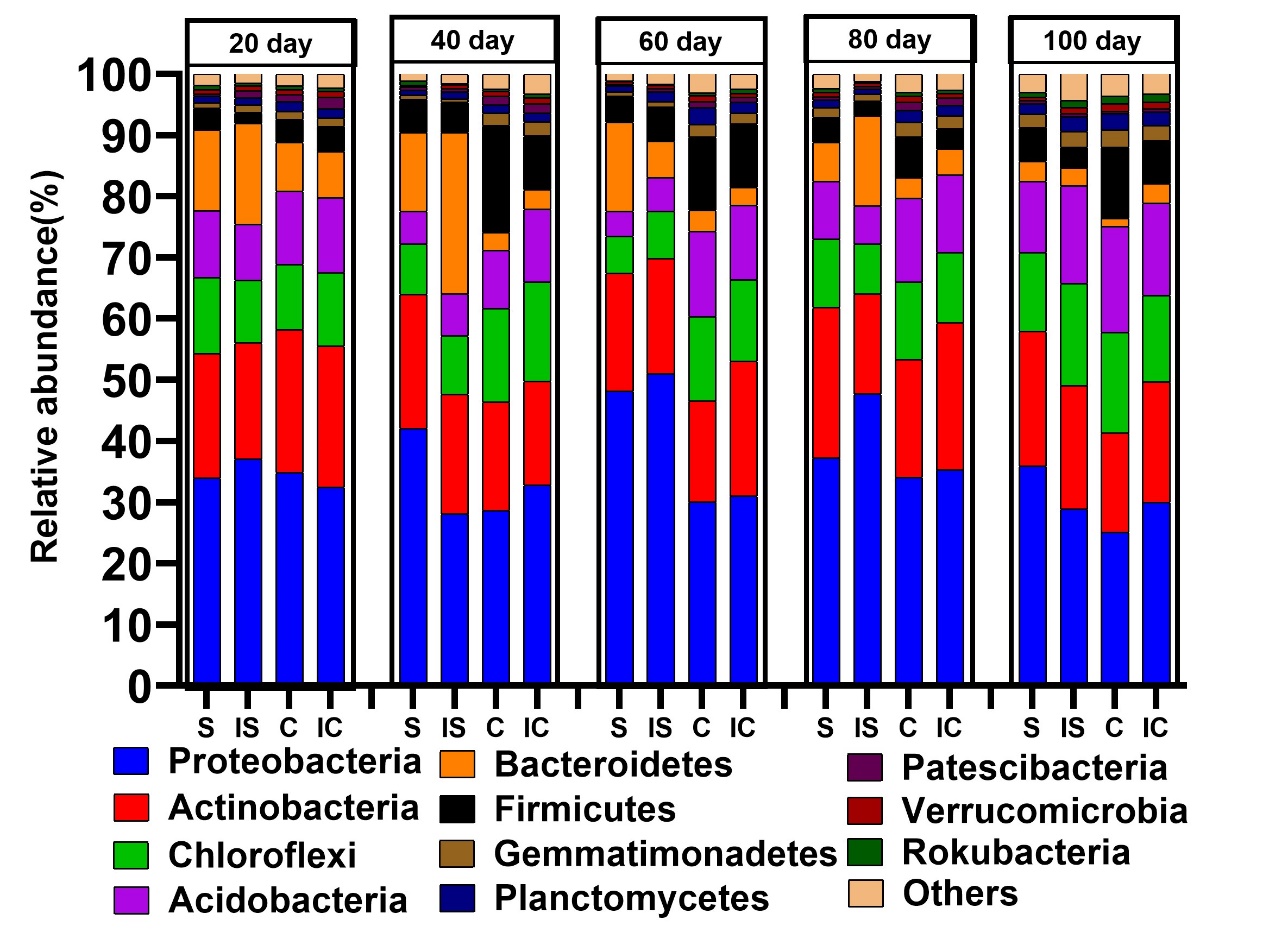


**Figure S3 The relative abundance of phyla at different time points under the two planting modes.** S: monoculture soybean soil, IS: intercropping soybean soil, C: monoculture corn soil, IC: intercropping corn soil.
