# Supplementary material for: Dynamic changes of rhizosphere soil bacterial community and nutrients in cadmium polluted soils with soybean-corn intercropping
Source: BMC Microbiol. 2022 Feb 15;22:57. doi: 10.1186/s12866-022-02468-3 (PMC8845239; doi:10.1186/s12866-022-02468-3)
Supplement: Supplementary file 4 — Additional file 4. [file 12866_2022_2468_MOESM4_ESM.docx]

**Table S3 Summary of module hubs in bacterial communities at five different time points.** S: monoculture soybean soil, IS: intercropping soybean soil, C: monoculture corn soil, IC: intercropping corn soil. Module hubs were nodes that highly connected with nodes within their modules, Zi > 2.5.

| **Group** | **Name** | **Phylum** | **Class** | **Order** | **Family** | **Genus** |
| --- | --- | --- | --- | --- | --- | --- |
| **S** | OTU_3 | Proteobacteria | Alphaproteobacteria | Rhizobiales | Xanthobacteraceae | Unclassified |
|  | OTU_16 | Actinobacteria | Thermoleophilia | Gaiellales | uncultured | uncultured bacterium |
|  | OTU_53 | Actinobacteria | Thermoleophilia | Solirubrobacterales | 67-14 | metagenome |
|  | OTU_65 | Acidobacteria | Acidobacteriia | Acidobacteriales | uncultured | uncultured bacterium |
|  | OTU_105 | Acidobacteria | Subgroup 6 | uncultured Acidobacteriales bacterium | uncultured Acidobacteriales bacterium | uncultured *Acidobacteriales bacterium* |
|  | OTU_111 | Proteobacteria | Gammaproteobacteria | Betaproteobacteriales | A21b | uncultured bacterium |
|  | OTU_188 | Proteobacteria | Gammaproteobacteria | Betaproteobacteriales | Burkholderiaceae | *Cupriavidus* |
|  | OTU_1600 | Firmicutes | Bacilli | Bacillales | Thermoactinomycetaceae | Unclassified |
| **IS** | OTU_3 | Proteobacteria | Alphaproteobacteria | Rhizobiales | Xanthobacteraceae | Unclassified |
|  | OTU_10 | Proteobacteria | Alphaproteobacteria | Rhizobiales | Methyloligellaceae | uncultured |
|  | OTU_14 | Proteobacteria | Alphaproteobacteria | Rhizobiales | Rhizobiaceae | *Allorhizobium-Neorhizobium-Pararhizobium-Rhizobium* |
|  | OTU_22 | Proteobacteria | Gammaproteobacteria | Betaproteobacteriales | SC-I-84 | uncultured bacterium |
|  | OTU_90 | Acidobacteria | Acidobacteriia | Solibacterales | Solibacteraceae (Subgroup 3) | *AKIW659* |
|  | OTU_134 | Proteobacteria | Alphaproteobacteria | Rhizobiales | KF-JG30-B3 | uncultured bacterium |
|  | OTU_152 | Proteobacteria | Alphaproteobacteria | uncultured | uncultured Bradyrhizobiaceae bacterium | uncultured *Bradyrhizobiaceae bacterium* |
|  | OTU_156 | Chloroflexi | TK10 | bacterium Ellin6519 | bacterium Ellin6519 | bacterium *Ellin6519* |
|  | OTU_164 | Acidobacteria | Acidobacteriia | Solibacterales | Solibacteraceae (Subgroup 3) | *Candidatus Solibacter* |
|  | OTU_236 | Bacteroidetes | Bacteroidia | Flavobacteriales | Flavobacteriaceae | *Flavobacterium* |
|  | OTU_242 | Acidobacteria | Acidobacteriia | Acidobacteriales | uncultured | uncultured bacterium |
|  | OTU_250 | Actinobacteria | Thermoleophilia | Gaiellales | uncultured | uncultured bacterium |
|  | OTU_550 | Bacteroidetes | Bacteroidia | Cytophagales | Spirosomaceae | *Spirosoma* |
|  | OTU_924 | Proteobacteria | Alphaproteobacteria | Acetobacterales | Acetobacteraceae | *Roseomonas* |
|  | OTU_1049 | Planctomycetes | Planctomycetacia | Isosphaerales | Isosphaeraceae | *Aquisphaera* |
|  | OTU_3305 | Proteobacteria | Alphaproteobacteria | Rhizobiales | Rhizobiaceae | *Aureimonas* |
| **C** | OTU_4 | Proteobacteria | Gammaproteobacteria | Pseudomonadales | Moraxellaceae | *Acinetobacter* |
|  | OTU_19 | Proteobacteria | Alphaproteobacteria | Rhizobiales | Rhizobiaceae | *Mesorhizobium* |
|  | OTU_39 | Nitrospirae | Nitrospira | Nitrospirales | Nitrospiraceae | *Nitrospira* |
|  | OTU_51 | Chloroflexi | KD4-96 | uncultured bacterium | uncultured bacterium | uncultured bacterium |
|  | OTU_118 | Proteobacteria | Gammaproteobacteria | Xanthomonadales | Rhodanobacteraceae | *Rhodanobacter* |
|  | OTU_130 | Proteobacteria | Alphaproteobacteria | Rhizobiales | Beijerinckiaceae | alpha cluster |
|  | OTU_299 | Acidobacteria | Acidobacteriia | Acidobacteriales | uncultured | uncultured forest soil bacterium |
|  | OTU_311 | Acidobacteria | Subgroup 6 | uncultured bacterium | uncultured bacterium | uncultured bacterium |
|  | OTU_434 | Proteobacteria | Gammaproteobacteria | Betaproteobacteriales | Burkholderiaceae | *Massilia* |
|  | OTU_438 | Bacteroidetes | Bacteroidia | Chitinophagales | Chitinophagaceae | uncultured |
| **IC** | OTU_39 | Nitrospirae | Nitrospira | Nitrospirales | Nitrospiraceae | *Nitrospira* |
|  | OTU_218 | Proteobacteria | Gammaproteobacteria | Betaproteobacteriales | Nitrosomonadaceae | *Ellin6067* |
|  | OTU_877 | Acidobacteria | Acidobacteriia | Solibacterales | Solibacteraceae (Subgroup 3) | *Bryobacter* |
|  | OTU_953 | Acidobacteria | Subgroup 22 | uncultured Acidobacterium sp. | uncultured Acidobacterium sp. | uncultured *Acidobacterium sp.* |
|  | OTU_1956 | Bacteroidetes | Bacteroidia | Chitinophagales | Chitinophagaceae | uncultured |
|  | OTU_2257 | Acidobacteria | Blastocatellia (Subgroup 4) | Blastocatellales | Blastocatellaceae | *Aridibacter* |
