# Supplementary material for: Dynamic changes of rhizosphere soil bacterial community and nutrients in cadmium polluted soils with soybean-corn intercropping
Source: BMC Microbiol. 2022 Feb 15;22:57. doi: 10.1186/s12866-022-02468-3 (PMC8845239; doi:10.1186/s12866-022-02468-3)
Supplement: Supplementary file 5 — Additional file 5. [file 12866_2022_2468_MOESM5_ESM.docx]

**Table S4 Summary of module connectors in bacterial communities at five different time points.** S: monoculture soybean, IS: intercropping soybean, C: monoculture corn, IC: intercropping corn. Module connectors were nodes that connected with several modules, Pi > 0.62.

| **Group** | **Name** | **Phylum** | **Class** | **Order** | **Family** | **Genus** |
| --- | --- | --- | --- | --- | --- | --- |
| **S** | OTU_54 | Acidobacteria | Acidobacteriia | Solibacterales | Solibacteraceae (Subgroup 3) | *Bryobacter* |
|  | OTU_163 | Proteobacteria | Gammaproteobacteria | Xanthomonadales | Rhodanobacteraceae | *Rhodanobacter* |
|  | OTU_173 | Acidobacteria | Acidobacteriia | Acidobacteriales | Acidobacteriaceae (Subgroup 1) | *Occallatibacter* |
|  | OTU_316 | Actinobacteria | Actinobacteria | Catenulisporales | Catenulisporaceae | *Catenulispora* |
|  | OTU_637 | Acidobacteria | Holophagae | Subgroup 7 | uncultured bacterium | uncultured bacterium |
|  | OTU_846 | Gemmatimonadetes | Gemmatimonadetes | Gemmatimonadales | Gemmatimonadaceae | uncultured |
|  | OTU_1344 | Gemmatimonadetes | S0134 terrestrial group | uncultured bacterium | uncultured bacterium | uncultured bacterium |
|  | OTU_1346 | Acidobacteria | Holophagae | Subgroup 7 | uncultured bacterium | uncultured bacterium |
|  | OTU_3224 | Proteobacteria | Alphaproteobacteria | Acetobacterales | Acetobacteraceae | uncultured |
| **IS** | OTU_27 | Chloroflexi | AD3 | uncultured bacterium | uncultured bacterium | uncultured bacterium |
|  | OTU_186 | Acidobacteria | Subgroup 6 | uncultured Holophagae bacterium | uncultured Holophagae bacterium | uncultured Holophagae bacterium |
|  | OTU_187 | Acidobacteria | Holophagae | Subgroup 7 | uncultured bacterium | uncultured bacterium |
|  | OTU_224 | Proteobacteria | Alphaproteobacteria | Reyranellales | Reyranellaceae | *Reyranella* |
|  | OTU_238 | Nitrospirae | Thermodesulfovibrionia | uncultured | uncultured Nitrospirae bacterium | uncultured Nitrospirae bacterium |
|  | OTU_247 | Acidobacteria | Subgroup 17 | uncultured soil bacterium | uncultured soil bacterium | uncultured soil bacterium |
|  | OTU_249 | Actinobacteria | Actinobacteria | Frankiales | Frankiaceae | *Jatrophihabitans* |
|  | OTU_342 | Bacteroidetes | Bacteroidia | Chitinophagales | Chitinophagaceae | *UTBCD1* |
|  | OTU_386 | Chloroflexi | Dehalococcoidia | S085 | metagenome | metagenome |
|  | OTU_688 | Proteobacteria | Gammaproteobacteria | Betaproteobacteriales | TRA3-20 | uncultured bacterium |
|  | OTU_930 | Gemmatimonadetes | Gemmatimonadetes | Gemmatimonadales | Gemmatimonadaceae | *Gemmatimonas* |
|  | OTU_3927 | Proteobacteria | Alphaproteobacteria | Sphingomonadales | Sphingomonadaceae | *Sphingomonas* |
|  | OTU_6713 | Actinobacteria | Thermoleophilia | Gaiellales | uncultured | uncultured bacterium |
| **C** | OTU_55 | Actinobacteria | Actinobacteria | Frankiales | Nakamurellaceae | *Nakamurella* |
|  | OTU_86 | Acidobacteria | Acidobacteriia | Solibacterales | Solibacteraceae (Subgroup 3) | *Bryobacter* |
| **IC** | OTU_4724 | Patescibacteria | Saccharimonadia | Saccharimonadales | Unclassified | Unclassified |
