# Supplementary material for: Dynamic changes of rhizosphere soil bacterial community and nutrients in cadmium polluted soils with soybean-corn intercropping
Source: BMC Microbiol. 2022 Feb 15;22:57. doi: 10.1186/s12866-022-02468-3 (PMC8845239; doi:10.1186/s12866-022-02468-3)
Supplement: Supplementary file 6 — Additional file 6. [file 12866_2022_2468_MOESM6_ESM.docx]

**Table S5 The relationship between the monoculture and intercropping corn bacterial community structure and soil properties. P-values were calculated using the distribution of the Mantel test statistics estimated from 9999 permutations.** *:P<0.05, **:P<0.01, ***:P<0.001.

| **Treatments** | **Factors** | **Shannon** | **Inv_Simpson** | **Observed_richness** | **Chao1** |
| --- | --- | --- | --- | --- | --- |
| **S-IS** | **pH** | -0.102 | -0.0521 | -0.255 | -0.263 |
|  | **SOM** | 0.519*** | 0.417** | 0.626*** | 0.588*** |
|  | **AP** | 0.186 | 0.202 | 0.31 | 0.297 |
|  | **AN** | -0.229 | -0.201 | -0.222 | -0.214 |
|  | **AK** | 0.268 | 0.31 | 0.38** | 0.366* |
|  | **ACd** | 0.158 | -0.0236 | 0.0595 | 0.00929 |
| **C-IC** | **pH** | 0.16 | 0.18 | 0.0651 | 0.139 |
|  | **SOM** | 0.0143 | 0.0285 | -0.106 | -0.0194 |
|  | **AP** | -0.137 | -0.144 | -0.19 | -0.0262 |
|  | **AN** | -0.254 | -0.246 | -0.244 | -0.18 |
|  | **AK** | -0.197 | -0.107 | -0.38* | -0.0395 |
|  | **ACd** | -0.0279 | -0.104 | -0.0149 | -0.00938 |
